# Artificial selection for improved energy efficiency is reaching its limits in broiler chickens

**DOI:** 10.1038/s41598-018-19231-2

**Published:** 2018-01-18

**Authors:** C. W. Tallentire, I. Leinonen, I. Kyriazakis

**Affiliations:** 1 Agriculture, School of Natural and Environmental sciences, Newcastle University, Newcastle upon Tyne, NE1 7RU United Kingdom; 2 Present Address: Land Economy Environment and Society Research Group, Scotland’s Rural College, Edinburgh, EH9 3JG United Kingdom

## Abstract

Modern broiler chickens are a major animal husbandry success story, both in terms of efficient resource utilisation and environmental sustainability. However, continuing artificial selection for both efficiency and rapid growth will be subject to both biological limits and animal welfare concerns. Using a novel analytical energy flow modelling approach, we predict how far such selection can go, given the biological limits of bird energy intake and partitioning of energy. We find that the biological potential for further improvements in efficiency, and hence environmental impact reduction, is minimal relative to past progress already made via artificial selection. An alternative breeding strategy to produce slower-growing birds to meet new welfare standards increases environmental burdens, compared to current birds. This unique analytic approach provides biologically sound guidelines for strategic planning of sustainable broiler production.

## Introduction

Livestock production systems impact considerably on the environment^1^. However, amongst different livestock systems, chicken meat production has been found to have relatively low environmental impacts^2,3^. This is in part due to artificial selection over the recent decades, aiming for increased energy use efficiency and faster growth rates^4–6^. As a result of increased growth rate, the birds reach their slaughter weight earlier than ever before. This has reduced the resource use of the bird, mainly because during the shorter growth cycle, less energy is now needed to maintain the body functions^7,8^. This improved energy efficiency has considerably reduced the feed consumption of the birds and therefore improved the environmental sustainability of broiler production.

The worldwide demand for chicken meat continues to grow substantially^9^; this is in part due to associated health claims, lack of cultural limitations on its consumption, the efficiency at which it is produced and human population growth^10^. The key questions are how this predicted increase in chicken meat production will be achieved, and what the consequences of this change on the sustainability of the production system will be. The poultry industry is confident that further improvements in growth rate and resource use efficiency can be achieved via genetic selection into the foreseeable future^11–15^. However, these predictions are not substantiated with biological evidence in the scientific literature, with some suggesting that growth rate will soon reach a maximum biological threshold that may be insurmountable with conventional breeding^16–19^. Industry data suggest that the actual rate of annual improvement in daily weight gain of birds has begun to decrease in recent years^4,5,20,21^. This may be partly explained by the changing objectives of artificial selection. Consumer concerns about the welfare of fast-growing chickens^22,23^ and their meat quality^24–27^, for instance, may have shifted selection pressures away from increasing growth rate in favour of other traits^28–30^ (e.g. robustness, reproduction and adaptability^31^). On the other hand, selection for increased growth rate will ultimately be subject to limitations dictated by the biology of the bird and, as a matter of course, a plateau will inevitably be reached. Such biological limits have not generally been considered by the poultry industry, when making predictions on the potential and the consequences of further genetic improvements of the birds in the future.

The growth of an animal is ultimately driven by the following thermodynamic processes: (1) energy (feed) intake, (2) transfer of the energy to the metabolic system (digestion), (3) loss of energy in metabolic heat production and (4) partitioning the chemical energy within the body. We construct a modelling framework based on evidence of the apparent biological limit of each of these processes to systematically analyse the potential for breeding for increased efficiency and concomitant changes in the associated environmental burdens. The environmental burdens considered are the greenhouse gas (GHG) emissions and agricultural land use (ALU) associated with feed production, as well as the excretion of nitrogen (N) and phosphorus (P); each of these indicators has potential implications on environmental impact (e.g. global warming, eutrophication and acidification) and food security. Our analysis shows that the physical limits of the biological processes determining bird growth are likely to be reached much earlier than currently predicted by the poultry industry. As a result, the potential to improve the environmental sustainability of broiler production through further artificial selection is limited. On the other hand, an alternative breeding strategy to produce slow-growing birds to meet expectations of improved animal welfare, via reducing growth rate so that slaughter weight is not reached until 56 days, will inevitably lead to increased resource use and therefore higher environmental burdens.

## Results

### Limits of feed intake

**Figure 1:**
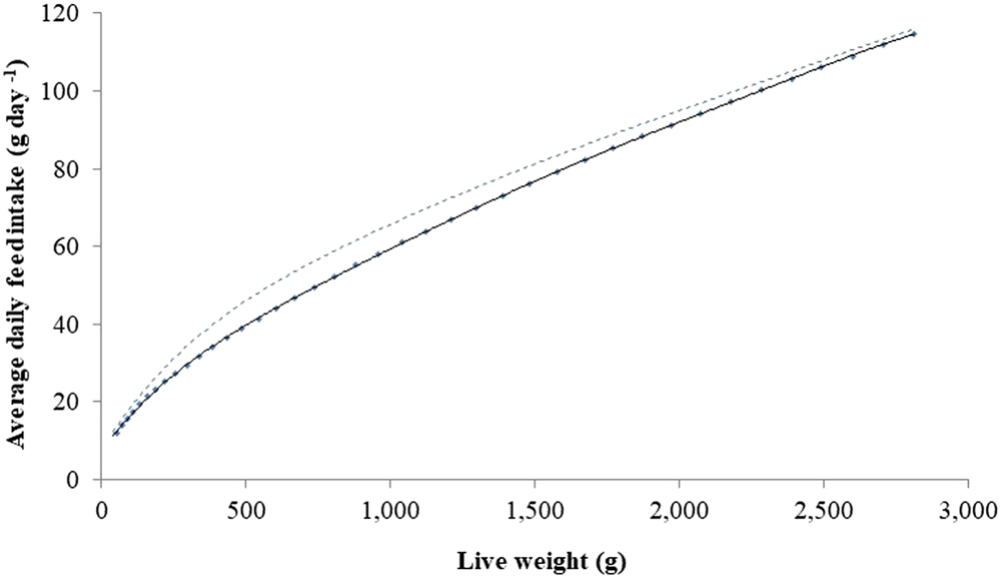
The average daily feed intake of a current fast-growing broiler () and the potential average daily feed intake defined by the apparent biological limit of feed intake (broken line). Based on the data presented by Leeson *et al*.^36^.

In order to increase broiler growth rate, and therefore increase the energy use efficiency towards the biological limit, the daily metabolizable energy (ME) intake must be increased to facilitate growth (Equation (1)). This can be achieved by increasing the daily feed intake and this has actually been the trend in commercial broiler breeding over recent decades^32–35^. In practice, this means that the birds must eat increasingly higher amounts at an increasingly younger age, which is biologically challenging. Ultimately, the theoretical maximum of feed intake would be set by the capacity of the digestive system. Experiments where the energy density of the feed was reduced (so the birds are forced to increase their feed intake) provide data on the fast-growing broiler feed intake limit^36–38^. The highest potential feed intake shown in literature was presented by Leeson *et al*.^36^ In that study, broilers increased their gross feed intake by a total of 25% upon reaching a live weight (LW) of 2.8 kg on a low energy content feed compared to a high energy feed fed control group. The potential daily feed intake can therefore be determined from these data. As an outcome, the average daily feed intake at a LW of 1.0 kg and 2.8 kg could be increased by 10% and 1.1% respectively compared to current fast-growing birds^21^ (Fig. 1). This indicates that younger birds have the greatest potential to increase feed intake which reduces as they approach slaughter weight (2.2 kg^39^). Although much genetic progress has been achieved since the study of Leeson, *et al*.^36^, more recently Linares and Huang^37^ showed that the feed intake of current fast-growing broilers could be increased by a further 6% between day 10 and day 42 when fed on a low energy content feed, compared to a high energy content feed. The limit to feed intake considered here is consistent with the latter study^37^.

### Limits of digestive efficiency

In artificial selection programmes, emphasis has been placed on the growth of certain body parts, such as the breast muscles, in order to increase carcass yield^5,34,40^. Consequently, the morphometries of the internal structures, in particular the organs that comprise the digestive system, have been shown to differ between high digestive efficiency genotypes and birds bred for high commercial performance^41,42^, i.e. increased energy use efficiency. In modern fast-growing birds, digesta throughput each day has increased to facilitate growth. Despite this, there is no evidence that breeding for increased commercial performance has led to any change in overall digestive efficiency per unit mass of digesta^7^; thus, selection pressure placed on increasing energy use efficiency and carcass yield at the very least must have conserved digestive efficiency whilst the size of the system has not increased at the same rate as other components of the body. Hence, the digestive efficiency as used in the energy flow model was expected to remain at its current level despite continuing selection for increasing energy efficiency. Since the digestible energy content of the feed per unit mass does not appear to be substantially compromised by augmented throughput^5,7^, nor does it appear to be improved genetically via selection for increased energy use efficiency^42–44^, it follows that the ME available to the broiler will be limited only by the capacity of feed intake.

### Potential changes in energy partitioning

Broilers currently have a body protein and lipid content of around 20% and 8%, respectively, based on recent data presented by Mussini^5^. The abdominal fat pad constitutes about 2% of the body weight^45,46^. Reducing this feature to zero would result in a bird with a body lipid content of around 6%. This value places the animal firmly at the lower end of the estimated biological limit for fatness^47,48^. Less energy is required to grow a leaner bird than a fatter bird at the same overall growth rate (Equation (1)). Therefore, reducing the body lipid content to its minimum redirects a higher proportion of the ME into the growth of the fat-free body components, thus allowing the bird to reach slaughter weight faster. As a result, reducing the fat content from the current level to the apparent biological limit would reduce the necessary energy intake upon reaching slaughter by 1.7% (Table 1).

**Table 1:** The effects of changing different processes of energy flow on the growth rate, total metabolizable energy (ME) intake and ME intake per unit mass of gain of a broiler grown to 2.2 kg.

Scenario	Age at 2.2 kg slaughter weight (days)	Growth rate (g day^−1^)	Total ME intake (MJ)	ME intake per unit gain (kJ g^−1^)
Current fast-growing broiler	34.2	63.1	45.9	21.3
Increased feed intake only	33.6	64.2	43.8	20.3
Increased leanness only	34.1	63.2	45.1	20.9
Increased feed intake and leanness (maximum energy efficiency breeding strategy)	33.0	65.3	42.0	19.4
Reduced growth rate and increased leanness (Increased welfare breeding strategy)	57.0	38.6	58.3	27.0

When changed, the feed intake and leanness are increased to their apparent biological limits.

In an earlier study, we found that the rate of metabolic heat production (*MHR*; MJ kg^−1^ d^−1^) of commercial broilers has either remained the same or been weakly positively correlated with the increase in growth rate^7^, over the recent decades, indicating that selection has not reduced the energy used for metabolic processes. Based on performance data for the current fast-growing birds^21^, the *MHR* was calculated to be 0.36 kg^−1^d^−1^. This same value was used to determine the energy distribution in the birds with maximum energy efficiency, as a conservative estimate for the further change.

### Predicted future broiler performance

**Figure 2:**
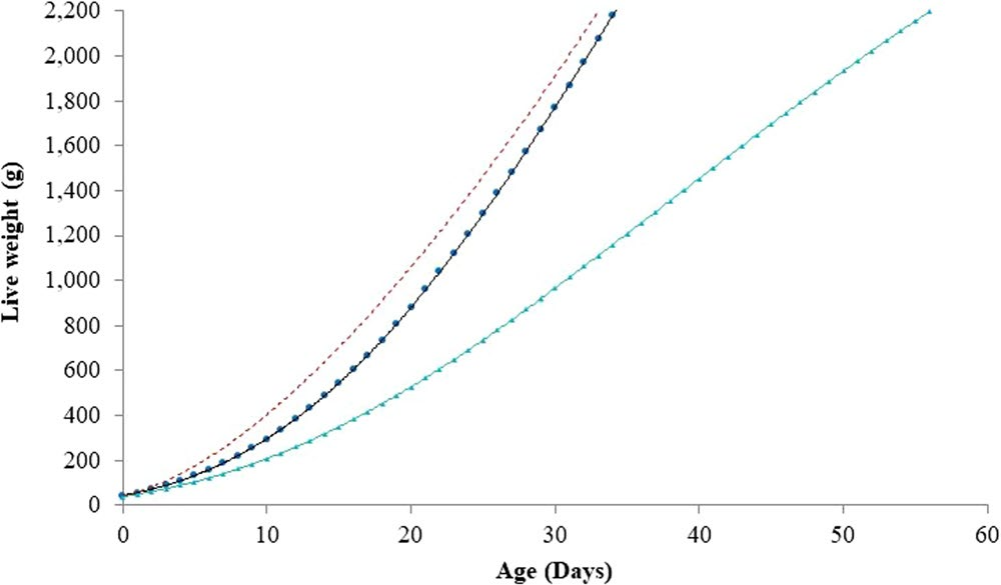
The growth rate of a current fast-growing broiler () and the potential growth rate of future birds as defined by the different scenarios accessed; maximum energy efficiency (broken line) and increased welfare scenario ().

The average age modern fast-growing broiler lines reach a LW of 2.2 kg (slaughter weight) is currently between 34 and 35 days of growth^21,49,50^. The outcome of our analysis shows that even if the broiler growth rate is increased to the apparent biological limit, this will result in birds that reach their slaughter weight only 1.2 days sooner (Fig. 2). This results in an 8% reduction in the total feed energy intake of the bird upon reaching slaughter weight (Table 1).

The analysis made above can be considered to represent a broiler bird with a maximum energy efficiency (and maximum growth rate). In an alternative scenario (increased welfare scenario), we calculated the energy intake of a slow-growing bird resulting from a higher welfare breeding strategy (i.e. growth rate is reduced so that birds reach a LW of 2.2 kg at 56 days of age, 23 days later than the current fast-growing bird). 5.7 MJ more energy per g of LW gain is required by birds from this slow-growing line to reach slaughter weight than is required by current fast-growing birds. This equates to 27% more total feed energy upon reaching slaughter than current fast-growing birds (Table 1).

### Environmental impact assessment of future breeding scenarios

**Figure 3:**
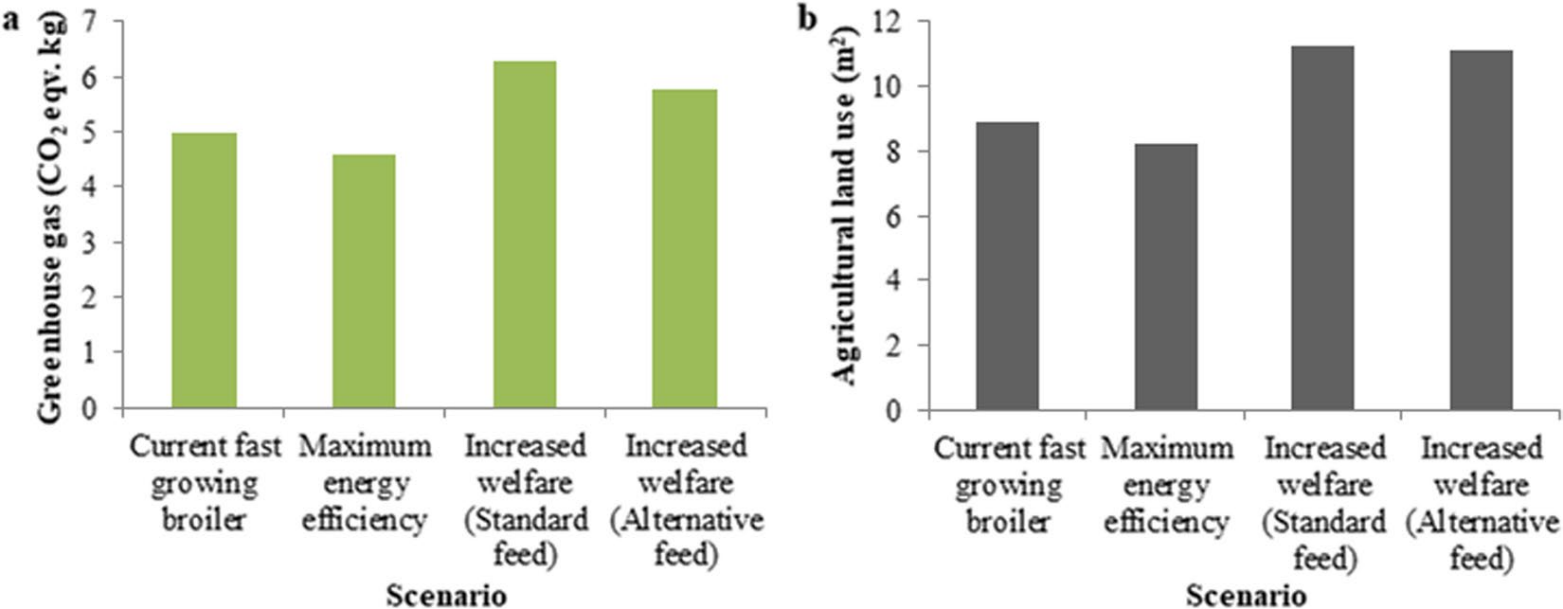
The environmental impact implications associated with feed provision for one broiler of each scenario grown to 2.2 kg. (**a**) shows greenhouse gas emissions (CO_2_ eqv.) and (**b**) shows the agricultural land use (m^2^). The following scenarios are presented: current fast-growing birds, maximum energy efficiency birds and slow-growing increased welfare birds placed on a standard feed, as well as slow-growing increased welfare birds placed on an alternative feed formulated specifically for the slow-growing line.

The maximum energy efficency scenario showed slightly reduced environmental burdens compared to current fast-growing birds, whereas the opposite was true for the scenario aiming to produce slow-growing “increased welfare” birds. The GHG emissions (Fig. 3(a)) and ALU (Fig. 3(b)) associated with feed production in the maximum energy efficency scenario were reduced by 8% when compared to current production. For the increased welfare scenario, both of these environmental indicators were increased by 27% when compared to current production on a standard feed. The environmental burdens were also calculated for the slow-growing line based on an alternative feeding programme (see Supplementary Table S1); this alternative feed had a lower protein content (19.6%) than the standard diet (21%), as to cater to the slow-growing line’s lower daily protein intake needed to maintain the required growth rate. When fed the alternative feed, GHG and ALU were increased by 16% and 24% respectively compared to current fast-growers reared on a standard feed (Fig. 3).

**Figure 4:**
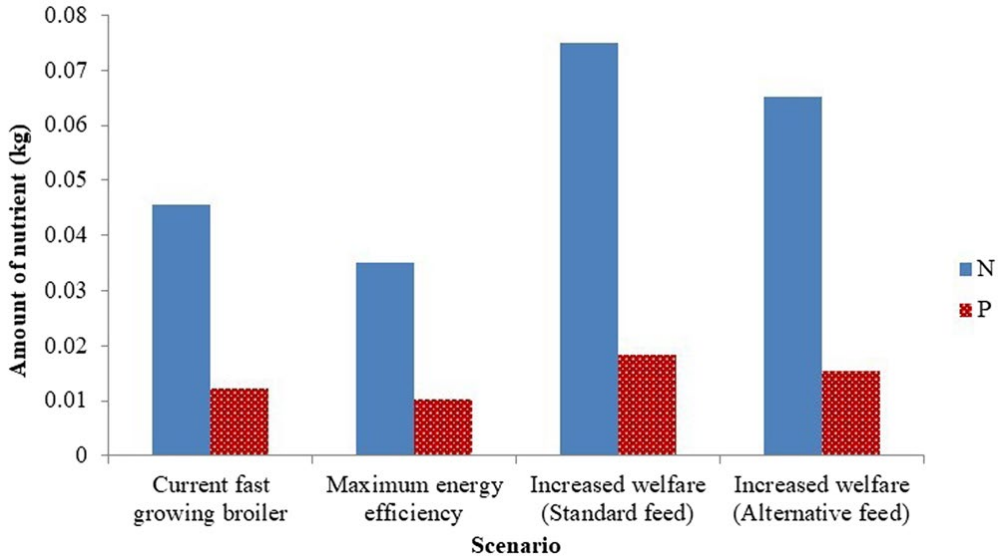
The nutrients, nitrogen (N) and phosphorus (P), that are expected to be excreted when one broiler is raised to 2.2 kg slaughter weight. The following scenarios are presented: current fast-growing birds, maximum energy efficiency birds and slow-growing increased welfare birds placed on a standard feed, as well as slow-growing increased welfare birds placed on an alternative feed formulated specifically for the slow-growing line.

The excretion of N and P were reduced by 23% and 15% respectively in the maximum energy efficiency scenario compared with current production, whereas these nutrients were excreted in higher quantities in the increased welfare scenario: an increase of 64% and 50% in the total N and P excretion was shown compared to current production on a standard feeding programme (Fig. 4). Applying the alternative feeding programme increased N and P excretion less than when the birds were raised on the standard feed, although this increase was still substantial (43% and 26% respectively).

Compared on a standard feeding programme with the maximum energy efficiency scenario, the slow-growing birds (increased welfare scenario) were associated with 37% more GHG and ALU, along with a 115% and 77% increase in N and P excretion respectively. When the alternative feeding programme was applied, with reduced feed protein content, the difference between the environmental burdens of the two future lines were reduced to 26%, 35%, 87% and 48% for GHG, ALU, N and P respectively.

## Discussion

Our results contrast with previous predictions^11–15^ and indicate that the biological potential for further improvements in energy efficiency of broiler production via artificial selection is low. Of the energy flow processes determining growth, we found no evidence that either the digestive efficiency or the *MHR* have changed as a result of recent artificial selection in a way that could improve the energy efficiency of the bird. In contrast, an earlier analysis shows that the *MHR* may have slightly increased (thus allowing less energy to be allocated to growth) during the recent decades^7^. Therefore, the current analysis may even overestimate the energy efficiency of the future bird. Overall, it would be very difficult to improve the bird energy efficiency by changing the *MHR* through artificial selection. In practice, this would require producing less active birds that use less energy for physical movement. This direction in future artificial selection is not very likely, taking into account the animal welfare concerns.

Reducing the carcass fat allows more energy to be allocated to the growth of fat-free body components. As the energy density of these components (consisting mainly of water and protein), is much lower than that of fat^8^, this allows the bird to be grown to a certain slaughter weight with lower energy intake. Although reducing the fat content of the body to its apparent minimum can improve the energy efficiency of the bird, this effect is rather small (Table 1) because the fat content of the current broiler birds is already low. This is due to their young slaughter age, and probably also due to continued artificial selection during the recent decades^6,51^. As a result, the only component of energy flow that can still substantially affect the bird growth and efficiency is the intake of energy. Therefore, the potential to increase the feed intake of the future bird largely determines the potential to increase its energy efficiency. Obviously, there are physical and biological limits in this process; although increased feed intake inevitably facilitates faster growth, it should be kept in mind that faster growing birds are also getting younger at the slaughter age, which sets limits to their feed intake capacity. The faster growth is largely allocated to certain parts of the body (e.g. breast muscle), and therefore the growth of the digestive system does not follow the increased growth of the bird as a whole^7^. The maximum feed intake capacity applied was based on the highest value found in literature for the current broiler bird^36^. Whilst increasing the feed intake further could facilitate even faster growth and more efficient birds, there is no evidence in literature to indicate birds can increase feed intake beyond this level. Furthermore, it should also be kept in mind that the maximum growth rate and energy efficiency can be achieved only if the voluntary feed intake of the bird is equal to the physical capacity of the digestive system or if the energy content of the feed were to be increased^52^.

The results show that even if the full potential of increasing growth rate and energy efficiency of the broiler birds is utilized, there is apparently very little room for improvement in the considered environmental sustainability indicators, relative to the total improvement in recent decades^5,6^ and when compared to earlier predictions of further selection^11,15^. For instance, when raised to a LW of 2.2 kg, a commercial broiler in 1978 could be estimated to be responsible for 25% more GHG and ALU associated with feed provision than a modern commercial fast-growing line^6^. In contrast, according to this study, the improvements still available to be made via artificial selection equate to only a further 8% reduction in GHG and ALU (Fig. 3). However, since we only compared the environmental burdens of growing one bird from each line to 2.2 kg, we did not consider any changes in carcass quality (e.g. white striping, woody breast and green muscle disease), which can occur with increased growth rate and breast muscle yield^25,27,34,53,54^ and lead to rejections at the meat processing stage. The need to produce a higher number of birds to replace the rejected meat would result in increased environmental burdens.

It is widely acknowledged that many instances of bird ill-health are associated with fast growth rate (e.g. musculoskeletal disorders, myopathies and organ failures)^55–60^. Hence, there has been a growing market demand for slow-growing broilers, which have perceived higher welfare, as an alternative to the fast-growing, energy efficient broilers^61–65^. The alternative future breeding scenario presented in this study followed the recommendation of ‘welfare-friendly’ policies adopted by some businesses across Europe^61^. Such policies stipulate that chickens must have a reduced growth rate and live a minimum of 56 days^54,62^. Growing this slow-growing line would result in a substantial increase of environmental burdens in every environmental indicator considered in this study due to its increased feed consumption. The difference in the environmental burdens between the two breeding scenarios was found to be slightly reduced when the slow-growing line was predicted to be fed the alternative feed, which had a reduced protein inclusion, instead of the standard feed. The alternative feed incorporated less soya meal, which is associated with high ALU relative to other crops (e.g. wheat and rapeseed) and high GHG emissions arising from land use change^66,67^. However, it should be noted that such an outcome is indicative only, as the future composition of the feed and the cultivation techniques of future feed crops are very difficult to predict.

This study demonstrates the apparent biological potential for environmental impact reduction in the poultry industry that is still available via artificial selection of broiler chickens. However, it is possible that the resource inputs into the broiler growing facilities could change in the future due to technological advancements and policy changes in animal welfare. Such changes will have environmental impact implications. Furthermore, producing birds that grow more slowly will also change the amounts of other resources spent on them besides feed, e.g. energy needed for heating of the growing facility and to power ventilation, lights and feed dispensers. The scenarios described in this study may also produce differences in mortality rates^34^; in conventional broiler systems, the proportional environmental impact of bird mortality is currently very low^67^ and is intrinsically linked to factors that may change in the future in order to optimise production efficiency or meet different welfare standards, such as stocking density (combined weight of birds allowed per floor area). Furthermore, despite there being no evidence that digestive efficiency can be increased via artificial selection for current performance objectives, the digestibility of feed ingredients could be increased in the future, e.g. via advancements in the understanding and application of exogenous enzymes and prebiotics^68^. The increased growth rate of broilers can only be facilitated by feed with high inclusions of highly digestible protein. In Europe, imported soybean meal is incorporated as the main protein source in broiler feeds (see Supplementary Table S1). The GHG emissions associated with chicken meat may therefore be mitigated in the future by incorporating protein alternatives to imported soybean, such as European grown soybean, microalgae or insect meal^69^.

This study shows that the potential to increase the environmental sustainability of broiler production through artificial selection for higher energy efficiency is low compared to what has been achieved in recent decades. It is the first time that the biological limits have been analytically considered and applied to predict the potential environmental consequences of breeding strategies in this way, despite the fact that there is a substantial interest in predicting the environmental impacts of future livestock scenarios^70,71^. These results raise important questions as to whether the magnitude of further reductions in the environmental burdens can justify the continuation down the route of artificial selection towards maximum energy efficiency, until the biological limits of the birds are reached. Such a breeding strategy may prove unsustainable for the industry considering the market shift in Europe in favour of slow-growing broilers. On the other hand, reducing the growth rate of the birds following consumer desire for increased bird welfare will unequivocally result in a less efficient bird with higher environmental impact than current fast-growers. Balancing these social, economic and environmental aspects of the sustainability of livestock production will continue to challenge the poultry industry in the foreseeable future. It is therefore in the industry’s interest to continue to pay close attention to both consumer demands and their associated environmental impact implications.

## Methods

### Energy flow model

**Figure 5:**
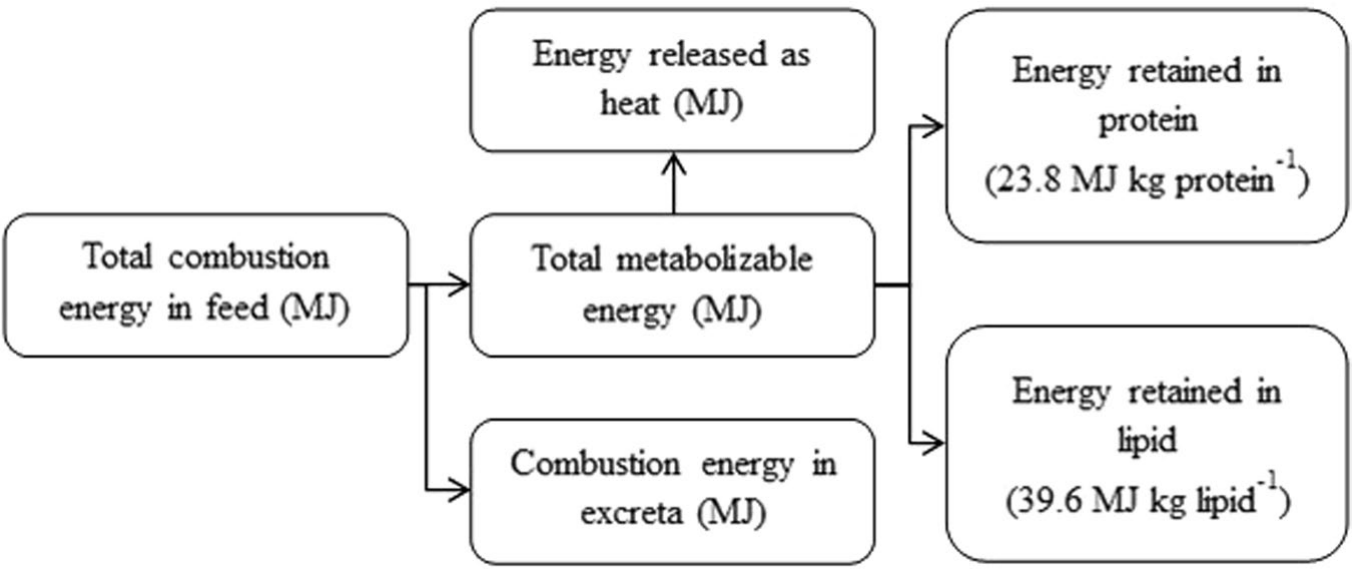
The components of energy flow through a broiler chicken.

We described the energy flow through the chicken body using a simple analytical model consisting of the following thermodynamic processes: (1) energy intake in the form of feed, (2) transfer of the energy to metabolic system, i.e. making a proportion of the combustible energy of feed utilizable in metabolic process through the process of digestion, (3) loss of energy in metabolic heat production, including all life-sustaining biochemical transformations within the cells, such as those related to physical activity, protein turnover and the maintenance of energetically expensive systems and (4) partitioning the chemical energy within the body, i.e. storing the energy in the form of lipid and protein (Fig. 5).

If the energy use efficiency is to increase further, and consequently the environmental impact of broiler systems is to reduce through reduced resource use, this will be achieved through changes in the above processes. Hence, the biological limits of efficiency were assessed with an analytical energy flow model, which was used to predict possible future broiler growth trends based on the apparent biological limits of these processes incorporated into the model.

The structure of the energy flow model is as follows (Fig. 5): The “gross” energy (GE) intake equates to the total combustion energy in the feed which is consumed by the bird. Increasing feed intake towards the bird’s apparent intake capacity automatically increases the GE intake rate (*GER*; MJ d^−1^). A proportion of this energy is not utilised by the bird and is lost in the excreta. The net energy intake which is available to the bird is hence referred to as the metabolizable energy (ME) intake. The ME intake rate (*MER*; MJ d^−1^) is thus determined by the coefficient of digestive efficiency (*D*_efficiency_); increased digestive efficiency will increase the amount of the GE that can be utilised by the bird and therefore increase the energy use efficiency (Equation (1)). The ME must then be distributed between what is stored, as protein and lipids, and what is lost as heat. The chemical energy retained in the body as protein and lipid can be quantified based on their heats of combustion, i.e. 23.8 and 39.6 MJ kg^−1^ respectively^8,72^. The overall fat-free body composition (i.e. water, protein and minerals) can be approximated based on allometric relationships^73^, and as a result, the combustion energy content of the body for birds with a certain body weight and a given fat content can be calculated. Since less chemical energy is stored in fat-free body components compared to lipid, the energy that is taken in can be used more efficiently for weight gain when leanness is increased. Energy is lost as heat through the metabolic processes related to lean and fat body growth, as well as other metabolic pathways, such as those essential processes for maintaining normal bodily functioning. Thus the total energy lost as heat is the ME intake minus the energy stored by the body in protein and lipids, and is accounted for by the coefficient for the *MHR*, which can be calculated on the basis of the total energy intake and the composition of the total LW (kg).

1 $$\begin{array}{rcl}MER[{\rm{MJ}}\,{{\rm{d}}}^{-1}] & = & GER[{\rm{MJ}}\,{{\rm{d}}}^{-1}]\,\ast \,{D}_{{\rm{efficiency}}}\\  & = & (23.8\,[{\rm{MJ}}\,{{\rm{kg}}}^{-1}]\,\ast \,{\rm{\Delta }}Protein[{\rm{kg}}\,{{\rm{d}}}^{-1}])\\  &  & +(39.6\,[{\rm{MJ}}\,{{\rm{kg}}}^{-1}]\,\ast \,{\rm{\Delta }}Lipid[{\rm{kg}}\,{{\rm{d}}}^{-1}])\\  &  & +\,(MHR\,[{\rm{MJ}}\,{{\rm{kg}}}^{-1}{{\rm{d}}}^{-1}]\,\ast \,LW[{\rm{kg}}]\end{array}$$

where:

*MER* = Metabolizable energy intake rate;

*GER* = Gross energy intake rate;

*D*_efficiency_ = Coefficient of digestive efficiency;

*MHR* = Rate of metabolic heat production;

*LW* = Live weight;

*ΔProtein* and *ΔLipid* = The daily increase of the protein and lipid mass, respectively.

To determine the coefficients *D*_efficiency_ and MHR, we went through the available literature on the energy efficiency of the current broiler lines, and the trends of their genetic changes over the recent decades (summarized by Tallentire *et al*.^7^). Based on this review, we could specify the values of these constants for the current, fast-growing birds and specify any possible changes in the current values compared to the historic data. If no past trends in these coefficients were found, we could expect that they are not affected by artificial selection, and will therefore remain unchanged also in the future breeding programmes.

### Daily feed intake and growth

We used literature on feeding experiments where the birds were forced to increase their feed intake, in order to determine their maximum daily feed intake capacity. This was then converted to *GER*, assuming that the composition of feed will remain unchanged (i.e. the production would be based on high-energy concentrate feed). The relationship between LW and the average daily feed intake for a current fast-growing bird was then quantified using a nonlinear curve^21,50^ (Fig. 1). This relationship is linear between 0.3 kg LW and the average slaughter weight of standard indoor broilers (2.2 kg^39^), therefore the potential feed intake each day beyond 0.3 kg LW was derived from the regression line equation for the maximum average daily feed intake limit each day presented in the literature^36^. As an outcome of this analysis, the daily live weight gain for the birds resulting from future breeding scenarios was calculated using Equation (1), with the expected values of GE, *D*_efficiency_ and *MHR*, and *ΔProtein* and *ΔLipid*, based on the expected changes in the body composition.

### Future broiler production scenarios

Two potential broiler breeding scenarios were addressed in this study. The first was based on the continuation of artificial selection for increased energy efficiency, as applied to current fast-growing lines reared commercially^21,50^. The performance of broilers subjected to further selection for increased energy use efficiency was calculated based on evidence of current genetic trends and apparent biological limits in the underlying biology^7^. In order to improve the energy use efficiency and increase the growth rate of the line further, the apparent biological limit of feed intake was applied to the current genotype, and the potential of the other energy flow processes (digestive efficiency, body composition and MHR) were changed to their apparent biological limits to facilitate this breeding strategy’s objectives. Finally, using the model shown in Equation (1), we specified the growth rate of the bird produced as an outcome of this scenario, and calculated the time and energy intake needed to reach the 2.2 kg slaughter weight.

In an alternative future breeding scenario, birds must be selected so that they reach slaughter weight no sooner than in 56 days^62,74^; this is also the minimum slaughter age currently required in free range chickens^65^. It is reasonable to assume, however, that for economic reasons the breeders will try to produce the most efficient birds possible within the limits of the welfare standard envelope, as well as the biological limits, via placing selective pressure on other traits than growth, i.e. body composition. Therefore, for the future slow-growing birds, we applied the following scenario: (1) We reduced the body fat content to its apparent biological limit. (2) We set the age when the slaughter weight is reached to be 56 days. (3) For other constants in Equation (1), we applied the same procedure as for the increased energy efficiency scenario described above. (4) Finally, we used Equation (1) to calculate the energy intake of the birds needed to reach a slaughter weight of 2.2 kg with a growth rate specified by the welfare standards. Hence, due to different selection strategy objectives, this procedure differed from that used in the scenario for the increased energy efficiency, where we specified the rate of energy intake according to biological limits of the bird only.

For the purpose of this study, the composition of the broiler feed was represented by two feeding programmes (see Supplementary Table S1). The first was based on the standard nutritional recommendations for current fast-growing birds^50,75^; this feed had an average energy and crude protein content of 13.2 MJ kg^−1^ and 21% respectively, and was here presumed to be fed to birds representing both of the future breeding scenarios; this was done in order to show the environmental implications of the artificial selection only. The second feeding programme was based on current nutritional specifications recommended for current slow-growing birds^76^; this “alternative feed” had a lower crude protein content compared to the standard feed and was fed to the increased welfare scenario only (see Supplementary Table S1). The composition of both feeds were formulated using a least cost formulation method, on the basis of current UK ingredient prices^77^. The feeds used in this study were, therefore, expected to be typical of current UK broiler production as a case in point for European systems. The primary energy ingredient of both feeds was wheat, whilst the main source of protein was provided by soybean meal, which is mainly imported to Europe from South America^66^.

### Environmental indicators

Energy provision (in the form of feed) represents the poultry industry’s greatest environmental hotspot^67,77^, hence the environmental indicators considered include inputs and outputs that are related to producing the feed required by one broiler bird to achieve a slaughter LW of 2.2 kg^39^. All upstream processes associated with feed production, such as resource inputs to fertilizer production and the emissions that arise as a result of their application to fields, as well as the energy inputs to processing and transport of ingredients, were based on current practices.

For the purpose of this study, the differences in the most relevant feed-related environmental indicators of broiler production were quantified. As such the environmental burdens of GHG emissions, measured in carbon dioxide equivalent (CO_2_ eqv.) with a 100 year timescale, and the ALU (m^2^), both associated with the feed provision, were calculated. The main agricultural sources of GHG are nitrous oxide (N_2_O) together with CO_2_ from fossil fuel and methane (CH_4_), although non-ruminant species produce negligible amounts of enteric CH_4_^2^. GHG emissions have impacts, such as global warming. 1 kg of CH_4_ and N_2_O emitted was considered to be equivalent to 25 and 298 kg of CO_2_ respectively^78^. The CO_2_ released due to land transformation was included following the PAS2050:2012-1 methodology^79^. The emission data for feed ingredients were based on national inventory reports, SimaPro databases and literature^77^.

The excretion of the environmentally important nutrients N and P were also considered as environmental indicators. Although the manure containing these nutrients can be used in the place of synthetic fertilizers (especially important in organic farming), excess of nutrients is associated with acidification and localised eutrophication, whilst N is responsible for the ammonia emissions at housing, manure storage and field spreading^78^. The N and P deposition in manure were calculated using the mass balance principle; the nutrients retained in the broiler’s body were subtracted from the total N and P supplied in the feed.

### Data availability

All data analysed during this study are publicly available and cited within this article. Furthermore, all data generated during this study are included within this published article (and its Supplementary Information files).

## Electronic supplementary material

Supplementary information
