# Publisher Correction: Artificial selection for improved energy efficiency is reaching its limits in broiler chickens

**DOI:** 10.1038/s41598-018-23133-8

**Published:** 2018-03-14

**Authors:** C. W. Tallentire, I. Leinonen, I. Kyriazakis

**Affiliations:** 1 Agriculture, School of Natural and Environmental sciences, Newcastle University, Newcastle upon Tyne, NE1 7RU United Kingdom; 2 Present Address: Land Economy Environment and Society Research Group, Scotland’s Rural College, Edinburgh, EH9 3JG United Kingdom

Correction to: *Scientific Reports* 10.1038/s41598-018-19231-2, published online 18 January 2018

The original PDF version of this Article contained an additional marker superimposed above Figure 1, which was erroneously introduced during the Production process. This has now been corrected in the PDF; the HTML version of the paper was correct from the time of publication.

