# Supplementary material for: Artificial selection for improved energy efficiency is reaching its limits in broiler chickens
Source: Sci Rep. 2018 Jan 18;8:1168. doi: 10.1038/s41598-018-19231-2 (PMC5773546; doi:10.1038/s41598-018-19231-2)
Supplement: Supplementary file 1 — Supplementary information [file 41598_2018_19231_MOESM1_ESM.pdf]

Artificial selection for improved energy efficiency is reaching its limits in broiler chickens

(Supplementary information)

C. W. Tallentire, I. Leinonen and I. Kyriazakis

Supplementary Table S1: Least cost broiler feed formulations, and their corresponding nutrient contents, typical of European production systems. The standard feed is formulated specifically for the requirements of current fast growing broilers. The alternative feed is formulated specifically for slower growing birds.

Ingredient	Standard feed	Alternative feed
Wheat (%)	47.9	51.2
Rapeseed (%)	6.7	7.1
Field peas (%)	12.3	13.2
Soy meal (%)	24.6	21.0
Soy Oil (%)	4.3	4.1
Minor ingredients and additives (%)	4.2	3.4
Nutrient content		
Metabolizable Energy (MJ kg^{-1})	13.2	13.2
Crude Protein (%)	21.0	19.6
*Dig. Lysine (%)	1.09	1.00
Dig. Methionine (%)	0.54	0.48
Dig. Methionine + Cystine (%)	0.84	0.77
Dig. Threonine (%)	0.73	0.67
Dig. Valine (%)	0.83	0.77
Dig. Isoleucine (%)	0.75	0.69
Dig. Arginine (%)	1.23	1.14
Dig. Tryptophan (%)	0.22	0.21
Dig. Leucine (%)	1.34	1.22
Available Phosphorous (%)	0.42	0.37
Potassium (%)	0.88	0.83
Calcium (%)	0.83	0.74
Chloride (%)	0.23	0.23
Magnesium (%)	0.17	0.17
Sodium (%)	0.16	0.16

*Dig = Digestible
